# Early Recognition of the Preference for Exclusive Breastfeeding in Current China: A Prediction Model based on Decision Trees

**DOI:** 10.1038/s41598-020-63073-w

**Published:** 2020-04-21

**Authors:** Yiting Wang, Chunjian Shan, Yingying Zhang, Lei Ding, Juan Wen, Yingying Tian

**Affiliations:** 1 Department of Obstetrics, Women’s Hospital of Nanjing Medical University, Nanjing Maternity and Child Health Care Hospital, Nanjing, 210004 China; 2 Nanjing Maternity and Child Health Care Institute, Women’s Hospital of Nanjing Medical University, Nanjing Maternity and Child Health Care Hospital, Nanjing, 210004 China; 3 Committee of Obstetrics and Gynaecology, Nursing Association of Jiangsu Province, Nanjing, 210004 China; 4 State Key Laboratory of Reproductive Medicine, Women’s Hospital of Nanjing Medical University, Nanjing Maternity and Child Health Care Hospital, Nanjing, 210004 China

**Keywords:** Epidemiology, Risk factors

## Abstract

Exclusive breastfeeding (EBF) is affected by multiple risk factors. Therefore, it is difficult for clinical professionals to identify women who will not practice EBF well and provide subsequent medical suggestions and treatments. This study aimed to apply a decision tree (DT) model to predict EBF at two months postpartum. The socio-demographic, clinical and breastfeeding parameters of 1,141 breastfeeding women from Nanjing were evaluated. Decision tree modelling was used to analyse and screen EBF factors and establish a risk assessment model of EBF. The Chinese version of the Breastfeeding Self-Efficacy Scale (CV-BSES) score, early formula supplementation, abnormal nipples, mastitis, neonatal jaundice, cracked or sore nipples and intended duration of breastfeeding were significant risk factors associated with EBF in the DT model. The accuracy, sensitivity and specificity of the DT model were 73.1%, 75.5% and 66.3%, respectively. The DT model showed similar or better performance than the logistic regression model in assessing the risk of early cessation of EBF before two months postpartum. The DT model has potential for application in clinical practice and identifies high-risk subpopulations that need specific prevention.

## Introduction

Breast milk is the safest and most nutritious food for infants^1^. Exclusive breastfeeding during the first six months, as well as the continuation of breastfeeding with additional food for two years or longer, is recommended by the World Health Organization (WHO) and United Nations Children’s Fund (UNICEF)^1,2^. Breastfeeding not only reduces morbidity and mortality but also has multiple long-term benefits for both children and mothers. For example, breastfeeding can reduce the incidence of obesity and non-communicable diseases and is consistently associated with higher performance in intelligence tests in children and adolescents^3–6^. The maternal benefits of breastfeeding include a more rapid return of postpartum uterine tone, postpartum weight loss, delayed resumption of menses, and decreased risks of breast, ovarian, and endometrial cancers^3–6^. Moreover, breastfeeding also strengthens the infant-mother bond and reduces or eliminates the cost of purchasing formula^6^. Despite the proven benefits of exclusive breastfeeding, many mothers cease the practice prematurely.

Breastfeeding is affected by a complex combination of factors, such as maternal sociodemographic factors, health care, psychosocial factors, type and availability of social support, and public policy. Numerous factors have been shown to influence the duration of exclusive breastfeeding (EBF) and the exclusiveness of breastfeeding, such as parity^7^, maternal education^7–9^, age^7,9^, employment^9–11^, smoking^12^, antenatal classes^7,13^, body mass index^14^, pacifier use^7,15^, and the intended duration of breastfeeding^9^. Psychological factors have also been linked to the duration of breastfeeding^16^. All of the breastfeeding factors are variable due to culture and perinatal period differences. Therefore, it is difficult for clinical nurses and breastfeeding professionals to identify women who will not practice exclusive breastfeeding well and provide effective interventions.

Breastfeeding in China has other unique features in addition to having a homogeneous population because Chinese society has undergone a rapid surge and transition. In China, 79.6% of infants were ever breastfed, and 20.8% of infants were exclusively breastfed at 6 months. However, only 11.5% and 6.9% continued to be breastfed at 1 and 2 years, respectively^17^. Jiangsu Province is one of the most economically developed coastal provinces in China. The urban population of Jiangsu Province reached 476.76 million, and its urbanization rate and economic aggregate are among the highest China. Additionally, Nanjing is the capital of Jiangsu Province, where the migrant population accumulates from all over Jiangsu Province. The acceleration of urbanization and economic development may have affected the practice of breastfeeding^18^. For example, due to the high housing price, young parents are forced to live with their parents. The influence of grandmothers on breastfeeding practice is mixed. Meanwhile, breastfeeding mothers must return to work early because of inadequate maternity protection in informal work sectors. The increased marketing of breast milk substitutes may have contributed to the decrease in breastfeeding.

The decision tree (DT) method is a data mining technology that has been widely applied in medicine and public health^19–22^. The DT model is a machine learning model comprising decision rules based on optimal feature cut-off values that recursively split independent variables into different groups to predict an outcome hierarchically. The decision tree can be used to transform a complex decision process based on the influences of different factors into a series of simple decisions^23^. The decision tree divides samples according to the best predictors of the results to provide risk assessments^19^. Furthermore, the DT method is a powerful statistical tool for classifying, predicting, interpreting, and processing data. The algorithm is non-parametric and can efficiently manage large, complex datasets without imposing a complex parametric structure. Missing values and heavily skewed data are easily managed without needing data transformation^24^. The computer-generated model is graphically represented as a tree structure that is easy to interpret and use in clinical settings compared with the OR values of the logistic regression (LR) model^25^.

The aim of this study was to investigate the risk factors involved in the early cessation of EBF and construct a prediction model based on a decision tree (DT) model that can provide a tool and improve clinical professionals’ ability to identify women at risk for discontinuing breastfeeding before two months postpartum. Additionally, the performance of the DT model was studied.

## Results

### Participant characteristics

**Figure 1:**
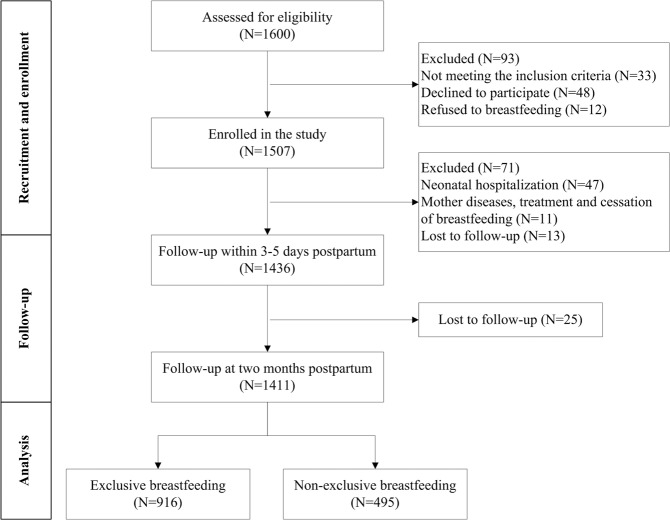
Flow diagram for the inclusion and exclusion criteria.

Among the 1,507 recruited participants, 1,411 women completed the study (retention rate = 93.6%). Meanwhile, 71 participants withdrew at 3–5 days postpartum, and 25 participants withdrew at the end of two months postpartum; the reasons for withdrawal are shown in Fig. 1. Among the 1,411 participants, 495 (35%) discontinued exclusive breastfeeding before two months postpartum. The rate of exclusive breastfeeding at two months postpartum was 64.9%. The socio-demographic, clinical and breastfeeding characteristics of the participants are presented in Table 1. Overall, the mean age of all the participants was 29.5 ± 3.49 (range:18–45) years. Most of the participants preferred to be primiparous (78.7%), had a vaginal delivery (63.7%), had a college degree (73.4%), worked full-time during pregnancy (88.7%), had a medium level of household income per year (75.5%), did not drink or smoke during the pregnancy (98.9%), lived with their parents (75.0%) and returned to work before 6 months postpartum (84.3%).

**Table 1:** Socio-demographic, clinical and breastfeeding characteristics of the participants.

Variable	Exclusive breastfeeding		OR	95% CI	P-value
	Yes, N = 916, (%)	No, N = 495, (%)			
Age, years	29.48 ± 3.41	29.51 ± 3.53	1.002	0.971–1.034	0.901
Gestation, weeks	39.66 ± 1.08	39.73 ± 1.06	1.060	0.957–1.175	0.265
Prenatal BMI, Kg/m^2^	25.97 ± 3.11	26.07 ± 3.08	1.010	0.975–1.046	0.593
Parity					
Primiparous	703 (76.7)	407 (82.2)	0.714	0.541–0.941	0.017
Multiparous	213 (23.3)	88 (17.8)			
Delivery mode					
Vaginal delivery	596 (65.1)	303 (61.2)	1.117	0.933–1.467	0.173
Caesarean delivery	320 (34.9)	192 (38.8)			
Maternal education					
High school or below	78 (8.5)	35 (7.1)	1.163	0.936–1.444	0.172
College graduate orbachelor degree	675 (73.7)	360 (72.7)			
Graduate or above	163 (17.8)	100 (20.2)			
Work full-time during the pregnancy					
Yes	802 (87.6)	450 (90.9)	0.704	0.489–1.012	0.058
No	114 (12.4)	45 (9.1)			
Household income, RMB/year					
<¥50,000	52 (5.7)	32 (6.5)	0.947	0.829–1.082	0.423
¥50,000–100,000	278 (30.3)	153 (30.9)			
¥100,000–200,000	411 (44.9)	223 (45.0)			
>¥200,000	175 (19.1)	87 (17.6)			
Living with parents					
Yes	697 (76.1)	361 (72.9)	1.181	0.920–1.516	0.191
No	361 (23.9)	134 (26.1)			
Drinking or smoking during pregnancy					
Yes	9 (0.98)	6 (1.21)	0.809	0.286–2.285	0.689
No	907 (99.02)	489 (98.79)			
Maternity leave time					
<4 months	207 (22.6)	115 (23.2)	0.902	0.807–1.049	0.211
4~6 months	558 (60.9)	310 (62.6)			
> 6 months	41 (4.5)	25 (5.1)			
Without work	110 (12.0)	45 (9.1)			
Intended duration of breastfeeding					
<6 months	85 (9.3)	78 (15.8)	0.927	0.867–0991	0.026
6~12 months	376 (41.0)	194 (39.2)			
>12 months	455 (49.7)	223 (45.0)			
Prenatal education attendance					
None	182 (19.9)	124 (25.1)	0.891	0.794–1.000	0.049
Few	372 (40.6)	193 (39.0)			
Almost all	231 (25.2)	116 (23.4)			
All	131 (14.3)	62 (12.5)			
Neonatal birth weight, g	3449.80 ± 379.82	3442.76 ± 373.27	1.000	1.000–1.000	0.738
CV-BSES scores	118.54 ± 19.44	100.27 ± 24.08	0.962	0.956–0.968	0.000
Early skin-to-skin contact and suckling					
Within an hour after birth	805 (87.8)	402 (81.2)	1.678	1.242–2.266	0.001
An hour after birth	111 (12.2)	93 (18.8)			
Using a pacifier					
Yes	24 (2.6)	19 (3.8)	0.674	0.365–1.243	0.207
No	892 (97.4)	476 (96.2)			
Perception of breast milk supply					
Oversupply	111 (12.1)	107 (21.6)	0.584	0.462–0.737	0.000
Normal	723 (78.9)	357 (72.1)			
Low supply	82 (9.0)	31 (6.3)			
Early formula supplementation					
Yes	278 (30.3)	248 (50.1)	2.304	1.839–2.887	0.000
No	638 (69.7)	247 (49.9)			
Abnormal nipples					
Yes	57 (6.2)	98 (19.8)	3.720	2.628–5.266	0.000
No	859 (93.8)	397 (80.2)			
Cracked or sore nipples					
Yes	123 (13.4)	93 (18.8)	1.492	1.111–2.003	0.008
No	793 (86.6)	402 (81.2)			
Mastitis					
Yes	140 (15.3)	123 (24.8)	0.546	0.416–0.716	0.000
No	776 (84.7)	372 (65.2)			
Neonatal jaundice					
Yes	183 (20.0)	151 (30.5)	0.569	0.443–0.731	0.000
No	733 (80.0)	344 (69.5)			

### Decision tree analysis

**Figure 2:**
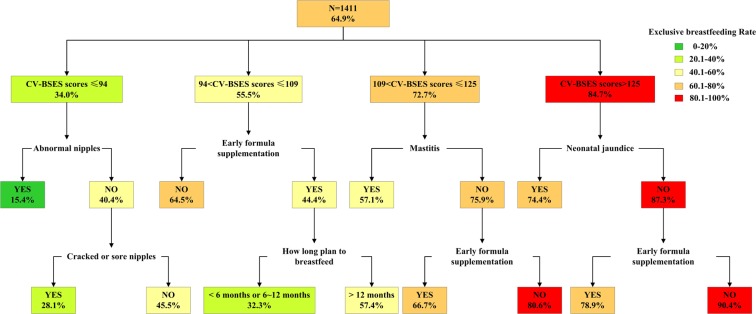
Decision tree predicting the risk for the early cessation of EBF at 2 months postpartum.

The decision tree resulting from Exhaustive Chi-squared Automatic Interaction Detection (exhaustive CHAID) analysis is shown in Fig. 2. This decision tree has a depth of four levels from the root node and 18 nodes, including 11 terminal nodes. Each node represents the probability of exclusive breastfeeding at two months postpartum for the corresponding branches. Based on the results of univariate analysis, ten significant predictors were found and were used to construct the decision tree model. Six significant predictors were successively selected to define further branches: the Chinese version of Breastfeeding Self-Efficacy Scale (CV-BSES) score, abnormal nipples, early formula supplementation, mastitis, neonatal jaundice, cracked or sore nipples and intended duration of breastfeeding.

The root variable in the decision tree is exclusive breastfeeding at two months postpartum. The variable that produces the first split in the decision tree is the CV-BSES score (χ^2^ = 216.981; p < 0.001; df = 3). At the second division, four branches appear in the decision tree: the first was abnormal nipples, followed by early formula supplementation, mastitis and neonatal jaundice. At the third division, cracked or sore nipples, intended duration of breastfeeding and early formula supplementation were important predictors associated with the risk of discontinuing exclusive breastfeeding prematurely. The tree shows that participants at a higher risk of discontinuing exclusive breastfeeding prematurely are those who have a low CV-BSES score (≤94) and abnormal nipples. The rate of EBF for these participants was only 15.4%. Additionally, those with a CV-BSES score >125, without neonatal jaundice, and who did not supply formula prematurely were more likely to continue exclusive breastfeeding at least two months postpartum. They also had the highest rate of exclusive breastfeeding (90.4%).

### Comparison between the DT and LR Models

To examine the performance of the DT model in predicting early cessation of EBF before two months postpartum, we compared it to the conventional LR model. The LR model included risk indicators that all appeared in the DT model—that is, the CV-BSES score, abnormal nipples, early formula supplementation, mastitis and neonatal jaundice. On the other hand, variables overlooked by the LR model, such as cracked or sore nipples and intended duration of breastfeeding, were important predictors of early cessation of EBF for certain subgroups in the DT model. The comparison of the performance parameters of the DT and LR models is presented in Table 2. The decision tree had an estimation risk of 0.278 and a standard error of 0.12, correctly classifying 73.1% of exclusive breastfeeding at two months postpartum. The sensitivity for this model was 75.5%, and the specificity was 66.3%. However, the accuracy, sensitivity and specificity of the LR model were 72.9%, 74.6% and 67.2%, respectively. The DT model had a better classification accuracy and sensitivity than the LR model.

**Table 2:** Comparison of the performance parameters of the logistic regression and decision tree models.

	Decision Tree Model		Logistic Regression Model	
	Predicted positives	Predicted negatives	Predicted positives	Predicted negatives
Diagnosed positives	796 (TP)	120 (FN)	809 (TP)	107 (FN)
Diagnosed negatives	259 (FP)	236 (TN)	276 (FP)	219 (FN)
Accuracy	0.731		0.729	
Sensitivity	0.755		0.746	
Specificity	0.663		0.672	

TP true positive, TN true negative, FP false positive, FN false negative a Accuracy = (TP + TN)/(TP + FP + TN + FN) b Sensitivity = TP/(TP + FN) c Specificity = TN/(TN + FP).

**Figure 3:**
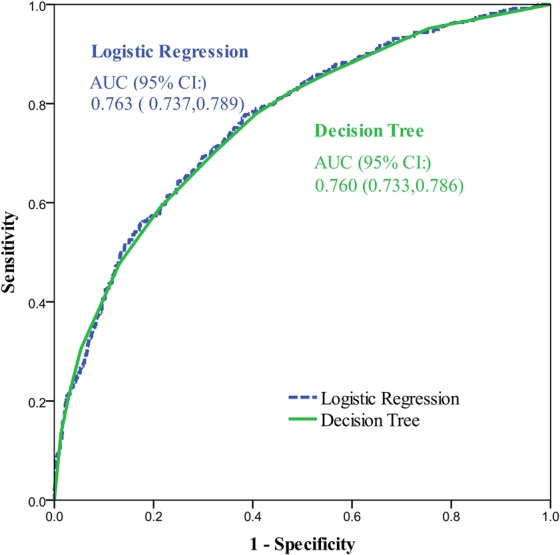
Receiver operating characteristic (ROC) curves for the decision tree and logistic regression models.

The area under the receiver operating characteristics (ROCs) curves (AUC) for the DT and LR models are shown in Fig. 3. The standard against which the sensitivity and specificity were calculated for both curves was EBF at two months postpartum. The areas under the curve were similar, 0.760 (0.733, 0.786) for the DT model and 0.763 (0.737, 0.789) for the LR model.

## Discussion

This study showed that the DT model can successfully identify women at risk for early cessation of EBF before two months postpartum and showed similar or better performance than the LR model. The decision tree method has some advantages in model construction and application. First, the algorithm can divide consecutive data into the best predictive group, efficiently segmenting populations into meaningful subgroups^20^. Additionally, high-risk population subgroups can be identified. Interventions can be developed according to the specific needs of each subgroup, especially for vulnerable groups. In this study, the group with a CV-BSES score lower than 94 was the most important target population, in which the EBF rate was only 34%. Additionally, the DT model described associations in the data by revealing important interactions among variables. Although abnormal nipples and cracked or sore nipples only had an effect on the group with a CV-BSES score lower than 94, they were meaningless in the higher CV-BSES score groups, indicating that the importance of each predictive variable varies in different subgroups. Furthermore, the DT model produced simple and visual tree graphics that can be easily applied in clinical practice. Therefore, DT can be easily used by clinical nurses and breastfeeding professionals.

The BSES score is one of the strongest predictors of early breastfeeding cessation. The maternal breast feeding self-efficacy is derived from the self-efficacy concept of Bandura^26^. According to Dennis, maternal breastfeeding self-efficacy refers to the mother’s perceived ability to breastfeed her child that affects her decision on breastfeeding, such as whether to breastfeed, how much effort to place on breastfeeding and how to respond to challenges during breastfeeding. Mothers with a high self-efficacy are more inclined to breastfeed, persist when confronted with difficulties, are self-motivated and react positively to challenges^27^. Thus, the BSES score can affect breastfeeding initiation, duration, and exclusivity, with a higher score indicating a higher rate of EBF and better performance in breastfeeding practices. This finding is consistent with findings from other cohort studies^28,29^. With each 10-point increase in the BSES score, the exclusive breastfeeding rate increased by approximately 12 to 20 percent in this study. Mothers who had a low score were more likely to give up exclusive breastfeeding prematurely when they faced difficulties, such as abnormal, cracked or sore nipples. Many studies have applied the BSES score to prenatal measurements and have suggested that it can significantly predict exclusive breastfeeding postpartum^30,31^. Thus, clinical nurses and breastfeeding professionals can use the BSES as a practical tool for prenatal and postnatal breastfeeding assessments, while the short form of BSES is more suitable and acceptable. Further research is warranted to provide more insight on this topic.

Based on the DT analysis, early formula supplementation is a widespread risk factor in many subgroups. In this study, 37.3% of neonates were supplied formula during hospitalization, a finding that is consistent with findings in previous breastfeeding studies in China^32,33^. The cause of this phenomenon is tightly related to the development status in China. With China’s accelerating urbanization, economic development, fast pace of life and other changes, breastfeeding practice has encountered many challenges. Particularly, the impact of the formula market should be seriously considered. Alarmingly, advertisements in the media have portrayed formula as being as good as, or better than, breast milk or as a lifestyle choice for the family. This misinformation can affect a mother’s decision to breastfeed^34^. It is easy to choose formula supplementation when mothers and their families perceive “inadequate breastmilk production” in the first week. Additionally, the convenience of shopping (online and offline) is another important reason. Presently, there are many ways to purchase formula, such as overseas online shopping, overseas purchasing agents, and domestic shopping websites. Indeed, China is now the largest infant formula market in the world, with a projected annual growth rate of 20 percent. Even in 2009, when the global real gross domestic product was negative, the annual sales of infant formula rose 8% in constant value^16^. In this situation, baby-friendly hospitals should play a role in protecting, promoting and supporting breastfeeding. Clinical nurses and breastfeeding professionals should enhance breastfeeding education from the prenatal to postpartum periods and include the benefits, knowledge and skills of breastfeeding.

In summary, clinical nurses and breastfeeding professionals should improve their ability to identify women at risk for discontinuing EBF before two months postpartum and target their efforts to provide practical help in clinical practice.

In this study, a DT model of EBF with relatively high sensitivity and specificity was established that not only identified independent risk factors predictive of an early cessation of EBF but also shed light on the assessment and management of EBF. Further validation of these models in a different database is required to test the accuracy of their classifications. Thus, work is in progress to gather new information at multiple centres and with heterogeneous populations to build a new dataset to test our models to predict early cessation of EBF.

## Methods

### Ethics statement

All participants were informed that their data concerning socio-demographic characteristics, clinical characteristics and breastfeeding practices that were collected would only be used for this study. A statement to the participants of our commitment to protect their personal information was also provided. All participants signed a consent form that declared their voluntary agreement with all of the procedures involved in this study in accordance with the tenets of the Declaration of Helsinki. Ethics approval for the study was granted by the Medical Ethics Committee of Nanjing Maternity and Child Health Care Hospital (File No.2016-112).

### Study population and study design

This population-based longitudinal study was conducted at a baby-friendly hospital with a delivery rate of 26,000 deliveries per year in Nanjing, China. Convenience sampling was used because of resource constraints from September 2016 to January 2017. Participants were recruited from the outpatient department of an antenatal clinic at 37 weeks of gestation. Participants were selected based on the following criteria: (1) were aged 18 years or older, (2) had a singleton pregnancy, (3) intended to breastfeed, (4) had no contraindications for breastfeeding, and (5) could communicate and complete the questionnaires. Some unqualified mothers were excluded due to the following: (1) they had a factor that could significantly interfere with breastfeeding, such as special treatment and medication, (2) they had a high-risk pregnancy (i.e., serious medical condition or known birth defect), or (3) the baby was not discharged home with the mother. Due to the large sample size recommended by data mining technology, this study planned to enrol 1,500 participants.

### Data collection at three stages

Potential predictors for breastfeeding were identified from published guidelines, systematic reviews and primary studies, including the three categories of socio-demographic characteristics, clinical characteristics and breastfeeding practices. According to the follow-up time, the predictors were measured by self-made questionnaires.

#### Time 1: 37 weeks of gestation

At 37 weeks of gestation at the antenatal clinic, participants were interviewed at a convenient location. After providing informed consent, eligible participants were asked by the research assistant to complete a socio-demographic questionnaire including their age, BMI, parity, education, employment status, maternity leave time, smoking and drinking behaviour, household income, and living and housing situation.

#### Time 2: follow-up within 3–5 days postpartum

All participants were interviewed in the postnatal ward after giving birth by a breastfeeding consultant at 3–5 days postpartum. Participants were asked to complete two-part questionnaires. Part A collected (1) clinical characteristics: delivery mode, perinatal complications, maternal medication use, and neonatal weight; (2) breastfeeding practices: prenatal education attendance, intended duration of breastfeeding, early skin-to-skin contact and suckling, pacifier use, early formula supplementation, abnormal nipples (flat, inverted or very large nipples), cracked or sore nipple, perception of breast milk supply, mastitis and neonatal jaundice. Part B was the Chinese version of Breastfeeding Self-Efficacy Scale (CV-BSES)^35^, which measured maternal breastfeeding attitudes and knowledge.

The CV-BSES is a 30-item self-report instrument that has been psychometrically tested using a population-based sample of 186 breast-feeding mothers in China. All of the items are preceded by the phrase ‘I am able to always …’ and are answered on a 5-point Likert scale ranging from 1 (not at all confident) to 5 (very confident). These ratings are summed into a total score ranging from 30 to 150, with a higher total score indicating a higher level of maternal breast-feeding self-efficacy. The Cronbach’s α coefficient of this instrument was 0.93, suggesting good internal consistency^35^.

#### Time 3: follow-up at two months postpartum

At two months postpartum, each woman was contacted by telephone to collect breastfeeding information by the research assistant. The main aim of this interview was to determine the mothers’ breastfeeding status. At the same time, the occurrence of cracked or sore nipples, mastitis and neonatal jaundice at two months postpartum were recorded. The time point of two months was chosen because the CV-BSES score was an important predictor in this study and proved to show good predictive performance for breastfeeding practice between 4 and 8 weeks postpartum^35^.

All of the research assistants and breastfeeding consultants who participated in the study were trained together and were given detailed instructions and explanations about the completion of questionnaires and outcome measures. A standardized interview schedule was used to maintain consistency.

### Statistical analysis

All data were analysed using the IBM SPSS 20.0 software package. Continuous data were compared using t-tests, and discrete count data were compared using χ^2^ test. Based on the results of univariate analysis, statistically significant variables were applied to construct a decision tree model and logistic regression model to assess the factors associated with breastfeeding cessation risk. In the DT model, the algorithm used was Exhaustive Chi-squared Automatic Interaction Detection or exhaustive CHAID. Chi squared test was used based on the minimum P value. Branching stopped when there were no risk indicators with a p-value less than 0.1 for division. The minimum sample size for each leaf (node) was specified as n = 50. The binary logistic regression method was used for multivariate analysis and establishment of the risk assessment model. An ‘entry’ approach was used to construct the LR model. A p value <0.05 was considered statistically significant. The performance of the predictive models was determined using AUC, accuracy, sensitivity and specificity. In both models, the predicted probabilities were the output and were used to create the curves. The higher was the AUC, the better performance the model predicted. The maximum AUC value is 1.0, indicating a perfect test. By contrast, an AUC value of 0.5 indicates no difference.
